# Exploring a green Swedish model: Coinciding and contradictory interests on a just climate transformation in Sweden

**DOI:** 10.1007/s13280-025-02144-6

**Published:** 2025-02-19

**Authors:** Jens Ergon, Roger Hildingsson, Mikael Karlsson

**Affiliations:** 1 Department of Earth Sciences, Uppsala University, Villavägen 16, 752 36 Uppsala, Sweden; 2 Department of Political Science, Lund University, P.O. Box 52, 221 00 Lund, Sweden

**Keywords:** Backlash, Green state, Just transition, Swedish model, Transformation, Welfare state

## Abstract

Climate policies are challenged around the world, a development coinciding with the backlash of globalisation. The emergence of the modern welfare state, however, indicates that similar tensions may give rise to transformative change, if coinciding interests for change develop. This article explores the case of Sweden, critical due to its longstanding role as an environmental frontrunner, now experiencing climate policy rollback. Based on 31 in-depth interviews, we analyse perceptions on a just transformation among business, trade union and civil society actors. The analysis reveals new and coinciding interests, driven by desire to restore climate leadership, and includes financial reform, more active state interventions and new policy tools to ensure public legitimacy. Views diverge on the depth of a just transformation, and power relations are different compared to the emergence of the welfare state. Nevertheless, a green Swedish model can be discerned, forging enhanced climate mitigation and renewed welfare state arrangements.

**Supplementary Information:**

The online version contains supplementary material available at 10.1007/s13280-025-02144-6.

## Introduction

In the run-up to the Paris Agreement, Sweden announced its ambition to become ‘one of the first fossil-free welfare nations’ (Swedish Government 2015). In 2017, the ambition was enshrined by a new climate act and a 2045 net-zero greenhouse gas emissions target, based on broad political agreement (Karlsson 2021), and in line with Sweden’s history as an environmental policy frontrunner (Lundqvist 2004). However, only five years later, the Swedish government has questioned adopted goals, cut environmental budgets, and weakened critical climate policy instruments, resulting in growing emissions and a historical break from past ambitions (SCPC 2024).

The rollback in Sweden is one of many examples where climate policies are met with growing resistance (Patterson 2023). The development has been described as a politicisation of the climate issue (Marquardt and Lederer 2022), including protest movements like the Yellow vests, elections of climate denying leaders such as Trump (Mendy et al. 2024), and deepening tensions about the future direction of climate policy in various regions (Patterson 2023).

These tensions may have many reasons. The climate crisis interlinks with a social trajectory of growing inequalities (Piketty 2014), democratic tensions (Streeck 2024) and increasing support for right-wing populist and nationalist parties (Scheiring et al. 2024). Sweden is a case in point of this development. Historically, Sweden has been regarded as an environmental pioneer and a recognised welfare state (Esping-Andersen 1990). However, while the Swedish environmental state (Duit 2016) has deepened in recent decades, the Swedish welfare model has eroded and transformed (Blomqvist and Palme 2020). Since the 1990s, socio-economic inequalities have grown more rapidly in Sweden than in many OECD nations, if from historically low levels in the 1980s (SFPC 2024). The development has been accompanied by eroding support for traditional parties and growing support for the radical right-wing Sweden Democrats (Rydgren and van der Meiden 2019). Before the 2022 national elections, three centre-right parties opened for collaboration with the Sweden Democrats (Aylott and Bolin 2023) and ran election campaigns criticising high prices on fuel and electricity, blaming Swedish climate and energy policy. When the elections resulted in a parliamentary majority, an agreement was made between the four parties (Moderaterna et al. 2022), which has resulted in a comprehensive dismantling of Swedish climate policy (SCPC 2024).

In this article, we use Sweden to investigate current tensions surrounding the climate transformation. We consider Sweden an interesting case, given its long history as an environmental policy frontrunner and parallel welfare erosion. The core is an empirical study of how key actors view the situation. We want to understand the reasons for these perceptions. Importantly, we note that the Swedish welfare model was built on a broad compromise (Blyth 2002), and explore whether and why similar types of compromises today might enable a fossil-free welfare state. The analysis combines concepts of just transitions (Krause et al. 2022), the emergence of the welfare state (Polanyi 2001), and the environmental state (Duit 2016). We conclude that a new situation has emerged in Sweden, in which key actors push for a green model, in response to the governments’ climate policy rollback.

The aim of the article is to understand the prospects for a green Swedish model and a just climate transformation, as perceived by key societal actors in Sweden. More specifically, we will answer the following research questions:

1. How do key societal actors in Sweden view the national rollback of climate policies?
2. What coinciding and contradictory interests do actors have in relation to a just climate transformation and what policy reforms do they consider important in this context?
3. What possible compromises can be identified between the actors?
4. What conflicts and interests risk being disregarded by such compromises?

The next section presents the literature underpinning our analysis. Section "Materials and methods" describes the interview and sampling methods. Section "Analysis" presents the results and our analysis. In Sect. "Discussion and conclusions", we conclude and discuss the analysis.

## Linking the welfare and environmental state to a just transformation

### Emergence and erosion of the Swedish welfare model

Esping-Andersen’s (1990) description of welfare regimes characterises the Swedish model by its universal nature and high degree of decommodification. The description builds on a Polanyian approach, viewing the post-war welfare states and embedded liberalism (Ruggie 1982) as outcomes of the Great Transformation (Polanyi 2001). Polanyi described this process as driven by a double movement, whereby expanding market forces were met by diverse countermovements. In the turbulent interwar era, developments gave rise to reactionary countermovements and totalitarian regimes. In Sweden, however, a transformative compromise managed to redirect such developments. While the electoral strength of the Swedish labour movement was a critical factor (Korpi 2006), so was the ability to forge coinciding interests for change, both among civil society, economic and political elites (Abrahamsson 2003). As described by Berman (1998), Swedish social democrats took a pragmatic route, emphasising societal alliances, as enshrined in the vision of ‘The People’s home’ (‘Folkhemmet’), alluding to both socially progressive and conservative sentiments. Swedish social democrats also embraced new economic policy, developing their own version of Keynesianism (Blyth 2002). The development was consolidated in 1938 by a historical agreement between the trade union confederation LO and Swedish employers (‘Saltsjöbadsavtalet’).

The historical compromise paved the way for a new hegemonic bloc, dominated for decades by social democrats and industrial elites. Notably, the compromise left property relations largely intact, stabilising a Swedish version of the Fordist growth models in the post-war era (Skyrman et al. 2023). An integral part was the so-called Rehn–Meidner model (Erixon 2010), through which Swedish trade unions affirmed the idea of creative destruction in return for social security, educational efforts, strengthened welfare, and a focus on full employment, all necessitating raised taxes and public investments.

Since the 1970s, however, the foundations of the Swedish model have changed substantially. The changes can be linked to the breakdown of embedded liberalism, increasing globalisation, and rise of neoliberalism (Cerny 2008). In Sweden, these processes emerged gradually in the 1970–1980s, but became manifest with the bank crisis in the early 1990s (Blyth 2002). The changes have involved substantial shifts in the political economy, including a resolution of the hegemonic bloc behind the post-war welfare model, towards a more neoliberal bloc, dominated by business elites (Ryner 2004). Economic development has changed towards a more financialised growth model (Belfrage and Kallifatides 2018), including deregulation of capital, lowered taxation, partial privatisation of public welfare, a restrictive fiscal policy framework, and a shift in focus from full employment towards price stability (Skyrman et al. 2023). Compared to the post-war era, the development has been characterised by higher unemployment rates and a substantial rise in income inequality and wealth concentration (SFPC 2024). While the Swedish welfare model has certainly preserved some of its characteristics, its universal nature has weakened (Blomqvist and Palme 2020), and its early high degree of decommodification declined (Skyrman et al. 2023).

### The emergence of the Swedish environmental state

Research on how states respond to environmental problems include theories exploring concepts such as the ‘green state’ (Eckersley 2004) and the ‘environmental state’ (Meadowcroft 2005), reflecting a normative ideal and an analytical object, respectively (Kronsell and Hildingsson 2022). The latter focuses on how modern states have gradually assumed more responsibilities to manage and regulate environmental change, a development that has contributed to the emergence of environmental states (Duit 2016).

The emergence of the environmental state is sometimes seen as an integral part of welfare state developments and some have emphasised analogies with the genesis of the welfare state (Meadowcroft 2005). One defining similarity is how the state is actively intervening to regulate markets and mitigate either social or environmental costs of economic development (Duit 2016). Connecting it to a Polanyian understanding of the development of the welfare state (Kronsell and Hildingsson 2022), the environmental state could thus be viewed as a result of a ‘green’ double movement, essentially between market forces and countermovements promoting environmental protection.

Sweden has often been seen as a pioneering environmental state (Duit 2016), developed at least since the Swedish Environmental Protection Agency was established in 1967. Being a frontrunner has long been a prime objective for environmental policy, by both left- and right-leaning governments. Over time, ideas of ecological modernisation (Mol and Spaargaren 2000) have fostered a predominant understanding, emphasising a win–win between environmental measures, industrial renewal and job creation. This has also applied to climate governance, as manifested in broad support for a carbon tax introduced already in 1991 (Hildingsson and Knaggård 2022), an energy transition towards renewable sources (Bäckstrand and Kronsell 2015), the recent climate policy framework (Karlsson 2021), and efforts aimed at industrial decarbonization (Hildingsson et al. 2019).

With its origin in the post-war era, the Swedish environmental state has been predicated on a neo-corporatist model of decision-making and negotiated governance (Lundqvist 2004). Albeit the Swedish model has transformed, similar kinds of relations are present in climate governance, however, until recently, with less participation from trade unions (Kronsell et al. 2019).

The kind of interventions in the economy have also changed, from an early focus on industrial command and control regulations towards more market-based instruments. Following the Paris Agreement and breakthroughs for renewable energy, decarbonisation has increasingly been perceived as a ‘race’ to promote ‘green growth’ and secure market shares in a fossil-free future (Lachapelle et al. 2017). State interventions have become both deeper and broader in scope, as exemplified by the EU Green Deal, the US Inflation Reduction Act (IRA), and trends of ‘de-risking’ (Gabor 2023). In Sweden, this includes new industrial policies supporting green transitions in for instance steel-making, not least in Northern Sweden, which experiences a new era of industrialisation, now with green connotations (Hildingsson et al. 2019).

Simultaneously, the consensus on environmental policy is challenged by increased politicisation (Marquardt and Lederer 2022) and polarisation of opinions on the climate issue (Axelsson and Jönsson 2023), driven in part by the rise of right-wing populism (Böhmelt 2021), but also by new climate movements (de Moor et al. 2021). In Northern Sweden, the green reindustrialisation gives rise to new environmental conflicts (Garbis et al. 2023). While Sweden has achieved absolute decoupling, emissions have decreased too slowly in relation to adopted targets, and decoupling does not involve material use (Haberl et al. 2020). Green state theorists have long pondered the ability of environmental states to pursue deeper green transformations (Eckersley 2021), in particular due to the economic imperative of the modern liberal state and its dependence on growth (Bailey 2018).

### Just transitions and transformations

The literature on just transitions and related concepts, such as climate justice (McCauley and Heffron 2018), is expanding fast (Wang and Lo 2021). Distinctions are made between distributional, procedural, and recognitional aspects (Heyen 2022). Importantly, tensions may exist between different perceptions (Ciplet and Harrison 2020). For instance, while dominating views of a just transition often focus on jobs and national distribution, views drawing on climate justice rather focus on global and intergenerational aspects.

Critical scholars have also emphasised a need to deepen the analysis of just transitions to include issues of power, clarifying questions such as ‘for whom?’, ‘towards what?’, and ‘by what means?’ (Newell et al. 2021). Notably, the aim of just transitions may vary widely, both in scale, scope and depth (Krause et al. 2022). Furthermore, ‘transition’ often designates a passage between two well-defined states, e.g. from fossil fuelled to decarbonised energy systems, whereas ‘transformation’ may be a more appropriate term for the society-wide changes likely necessary to reach the goals of the Paris Agreement (Linnér and Wibeck 2019).

In this article, we combine concepts of the welfare state, environmental state and just transition to analyse perceptions of a just transformation among Swedish actors. Notably, the starting point is an environmental state subject to climate policy rollback, and a partly eroded welfare state. While focus is put on a national level (scale), a just transformation may have different scope (involving both the environmental and welfare state, i.e. both environmental and social relations) and depth (level of change in the political economy, and decommodification). Building on Krause et al. (2022), we distinguish between reforms depending on scope and depth, as either continuing current climate policy rollback and welfare policy retrenchment, being managerial (aiming to stabilise the political economy), structural (modifying the political economy, i.e. away from neoliberalism), or deep structural (changing the political economy more deeply, e.g. towards deeper decommodification of nature). The idealised types are summarised in Table 1. It should be noted that reality is more complex and that policy measures can have both, for example, managerial and structural aspects.

**Table 1:** Ideal types of reforms according to depth (vertical) and scope (horizontal). Developed from Krause et al. (2022)

Type	Characteristics	Environmental state	Welfare state
Rollback	Driven by backlash, retrenched environmental and welfare states	Ignored near term climate targets, dismantled climate policy tools, reduced environmental budgets, intensified commodification	Stricter migration rules, less universal welfare provisions, tax reductions, austerity, continued commodification
Managerial	Driven by crisis management aiming to stabilise political economy	Reaffirmed climate goals, weak de-risking, limited green investments, market-based climate instruments, limited decommodification	Targeted just transition measures rather than universal protection, no substantial increase in public spending, limited decommodification
Structural	Modified rules of political economy and role of the state, i.e. away from neoliberalism	Interventionist state, financial reform, robust de-risking, industrial policies, substantial green investments, new climate instruments, significant decommodification	Strengthened welfare state, financial reform, substantial increase in spending on education and social infrastructure, strengthened social security, significant decommodification
Deep structural	Deeper changes in political economy, e.g. away from growth paradigm	Caps on production or consumption, reforms towards circular economy, post-growth measures, deep decommodification	Universal welfare provisions, raised taxes on capital and wealth, reduced working times, deep decommodification

## Materials and methods

The empirical material in this case study primarily consists of semi-structured interviews. Sweden is a pertinent case to study. First, it exemplifies a general trend of climate policy backlash, including possible interactions between strengthened climate ambitions, welfare erosion and growing support for right-wing populism. Second, it is of general interest to explore whether a long frontrunner tradition—with increasingly comprehensive policies, since the emergence of the Swedish environmental state in the late 1960s—influences the views on a just climate transformation. Studying how key actors view rapid policy dismantling in a frontrunner country can give important insights on climate policy backlashes elsewhere.

### Sample frame

We use purposive sampling to complement the historical actors behind the Swedish model with broader segments of civil society organisations (CSOs).^1^ We focus on civil society and business elites, leaving political elites to further inquiries. The actors are divided into three main categories (see Table 2). Thirty-five actors were sampled: 11 in category 1, 10 in category 2, and 14 in category 3, of which 31 participated (see Appendix S1).

**Table 2:** Categories and subcategories of sampled actors

Category 1: Trade unions	Category 2: CSOs	Category 3: Business organisations
1 blue-collar trade union confederation	6 environmental CSOs, stretching from large, mainstream CSOs to youth climate movement	1 employers’ confederation representing large firms
2 white-collar trade union confederations	2 CSOs representing indigenous people	1 employers’ confederation representing small and medium firms
6 blue-collar trade unions, representing private and public employees in different sectors	1 CSO representing rural interests	1 farmers’ confederation
2 white-collar trade unions, representing private and public employees in different sectors	1 CSO representing the tenants’ movement	4 employers’ organisations, representing different sectors and capital interests
		7 industrial organisations

### Data collection

In-depth interviews were carried out from 8/11 2023 to 9/1 2024. Interviews lasted 70–120 min and were carried out in person or by Zoom and recorded digitally. Whenever possible, interviews were carried out with elected leaders, CEOs or official spokespersons. Thus, data cannot give deeper insights on potential internal differences within organisations. Interview methods table, questionnaire and coding process are described in Appendix S1–S3. The study has been ethically approved.

## Results

### General perceptions: Sweden as a frontrunner

Overall, our material points towards a surprisingly broad overlap among a majority of the respondents on several key issues regarding Swedish climate policy. This includes a widespread dissatisfaction with current dismantling of climate policies and calls for stronger political leadership. We find nearly unanimous support for the Swedish climate goals and Sweden acting as a frontrunner.

Perceptions of current policy changes range from strong disappointment to concerns over political uncertainty and lack of leadership. A large trade union actor summarises prevalent views among many CSOs: ‘The current policy is deplorable. We are moving backwards into the future, with […] and absolutely no political leadership.’ While business actors are more restrained, they share many concerns: ‘It’s too bad that Sweden doesn’t act as the leader we had hoped for. […] It’s a totally wrong turn. To make investment decisions you need political stability’, argues a large employers organisation. Another employers’ organisation adds: ‘Take the biofuel mandate. The forest industry and others have invested a substantial amount of money to get these biofuels. And now they suddenly say, no, we don’t want this anymore’.

To restore Sweden’s status as a frontrunner, many actors call for political leadership, including a more active, interventionist state, taking the beat stick in the climate transformation, but also implementing tools to distribute costs and benefits and ensuring legitimacy and public acceptance: ‘We want the state to take on an increased responsibility for the gigantic structural transformation of the society that this is all about.’, says one industrial respondent. ‘I would like to hear the prime minister stating that Sweden will be a frontrunner, that we will go further than the Paris Agreement’, states a respondent of a trade union confederation.

Demands for a more interventionist state among business, and for climate leadership among trade unions, are relatively new positions. While motivations for restoring Sweden’s frontrunner position differ, many actors mention capability, responsibility, and frontrunner advantages: ‘Sweden has the privilege to be a frontrunner. We have the social structures, the technology, the economy, everything that is needed… It is a moral duty… Besides, it’s about egoism. If Sweden should be competitive we need to be a frontrunner’, one trade union confederation argues, echoing arguments behind the Swedish model. Environmental CSOs emphasise Sweden’s responsibilities and capabilities: ‘It is very important from a justice perspective. We have been among those who have emitted the most. We also have the capacities many other nations lack’. While business actors differ on the leadership-role in relation to EU policies, they forge arguments of advantages and responsibilities: ‘From our perspective it is incredibly important that we live up to what has been agreed. It creates benefits for the environment and increases the competitiveness of Swedish industries’, states a respondent of a large employers’ organisation. The revealed overlap between these different interests signify a new situation in the Swedish climate debate, contrasting the political development. Rather than polarised, actors seem united in their calls for political leadership.

### Coinciding interests, different reasons

It should be emphasised that coinciding interests may be temporary and are not the same as shared general interests. Motives and visions vary considerably between actors, manifested in different views on the scale, scope and depth of a just climate transformation.

A key driver for business is a perceived global race towards decarbonisation, in which Swedish industries may capture large market shares. The transformation is viewed as an opportunity, delegitimization and political uncertainty as risks. Demands for strong and stable climate policies, including a more active state and measures to ensure public acceptance, are motivated by such arguments: ‘We are in the starting blocks. And we have enormous advantages, geographically, from natural resources. […] If we handle this right’, argues one large employers’ organisation.

Among trade unions, positions have changed. If the climate transformation was formerly considered a global issue, of limited importance, it is increasingly viewed as an ongoing structural transformation, affecting union members at work and in their daily lives. ‘There are both challenges and opportunities. But with the right political will, there are almost only opportunities. […] The challenge is to make people feel secure, and that nobody feels that they only lose’, argues a respondent of a central trade union confederation.

Environmental CSOs are primarily motivated by climate change as a planetary crisis and foremost view the transformation as a necessity and a moral obligation: ‘The opportunity is that we avoid all the very harmful consequences that may come with climate change’, states a large environmental CSO. Strong climate policies are thus essential, but also measures to ensure legitimacy: ‘The challenge concerns participation and democracy. To bring people along’.

Among CSOs representing indigenous (Samí) people, the local effects of climate change are central: ‘We have adapted to climate change for thousands of years […] But nature is changing with an incredible speed today’. Simultaneously, strong fears are raised of the environmental effects of current modes of decarbonisation. ‘One of the worst images I have was when [the energy minister] said: “Now no birds nor frogs will stand in the way for big… industrial projects.” This is terrible news for us.’

The detailed picture is thus more complex, with tensions and differences. Within business, the government’s emphasis on electrification is welcomed, but respondents are ambivalent to its focus on nuclear power, and directly critical to sudden shifts in goals and instruments. Calls for strong and stable climate policies are most pronounced among industry organisations, including energy, technical and basic industries, while outspoken critics diminish at higher levels and among some business actors. For instance, we find a clear distinction between large industrial interests and small- and medium-sized enterprises (SMEs). The SME actor stands out, being most negative towards stricter climate policies and explicitly supportive towards current policy changes.

Among trade unions, blue-collar unions emphasise distributional justice and view a re-strengthened welfare state as integral of a just transformation, while white-collar unions are more cautious on equality demands. Notable differences also exist between sectors. Public sector unions and retail workers are more engaged in sustainable consumption, while trade unions in private and industry sectors focus more on production, jobs and investments.

Environmental CSOs are substantially more critical to the prospects for green growth than trade unions and business organisations. The youth climate movement and Samí actors stand out in this regard. While the largest overlaps in the material are found between trade unions and industry organisations, the biggest tensions are found between business organisations, Samí actors and the youth climate movement.

### Perceptions and demands on a just transformation

While actors differ on their perceptions of a just climate transformation, we find broad coinciding interests on several key issues and policy demands (see Table 3).

**Table 3:** Key policy demands, scope, depth, and supporting actors. ES, environmental state; WS, welfare state

Policy demand	Scope	Depth	Actors
Political leadership	ES	–	Broad support
Reaffirmed climate goals	ES	Managerial	Broad support
More active state interventions	ES/WS	Structural	Broad support
Increased public spending	ES/WS	Structural	Broad support
Reformed financial framework	ES/WS	Structural	Broad support
Investments in power and electrification	ES/WS	Managerial	Broad support
Investments in railways	ES/WS	Structural	Broad support
Compensation schemes for wind power	ES/WS	Structural	Broad support
New policy tools to redistribute costs	ES/WS	Structural	Broad support
Investment subsidies to green technologies	ES/WS	Managerial	Broad support
Reformed road tax and travel deduction	ES/WS	Managerial	Broad support
Increased spending on education	WS	Structural	Broad support
Investments in social structures	WS	Structural	Broad support
Faster permit processes	ES	Managerial	Business org, trade unions
Green investment bank	ES	Structural	Trade unions, industry org
Investments in public transportation	ES/WS	Structural	Trade unions, CSOs
Climate requirements on public procurement	ES/WS	Structural	Trade unions, CSOs
Subsidised energy efficiency programs	ES/WS	Structural	Trade unions, env CSOs, tenants’ CSO
New instruments towards circular economy	ES	Deep structural	Trade unions, env CSOs, Sami CSO
Subsidised public transportation	ES/WS	Structural	Trade unions, env CSOs
Strengthened active labour market policies	WS	Structural	Trade unions, env CSOs
Strengthened unemployment insurance	WS	Structural	Trade unions, env CSOs
Raised taxes on capital, wealth, high incomes	WS	Deep structural	Blue-collar unions, some CSOs
Reduced working time	WS	Deep structural	Env CSOs, some trade unions
Phase-out of fossil fuel subsidies	ES	Structural	Env CSOs, some trade unions
Support for biodiv protection and carbon sinks	ES	Structural	Env CSOs, some business org
Access to finance for SMEs and rural regions	ES/WS	Structural	SME org, farmers org, rural CSO
Improved dialogues in permit processes	ES	Managerial	Env CSOs, Sami CSO, rural CSO
Simplified environmental reporting to EU	ES	Managerial	SME org, farmers org
Tighter climate goals	ES	Structural	Env CSOs, Sami CSO
Reforms for less intensive forestry	ES	Deep structural	Env CSOs, Sami CSO
Legal rights to nature	ES	Deep structural	Sami CSO, youth climate movement
New instruments for reduced flying	ES	Deep structural	Env CSOs
Meat tax	ES	Structural	Env CSOs
Differentiated VAT	ES	Structural	Env CSOs

Among trade unions, ‘security under transformation’, ability to participate and a just distribution of costs and benefits are perceived as central: ‘The challenge is to make people feel secure, able to participate, and by that also feel pride in the transformation’, argues a respondent of a large trade union confederation. Many union actors refer to the Swedish model, arguing that Sweden has been a champion in handling just transitions in the past, and could be it again, given the right political measures and leadership. A common view, in particular among blue-collar unions, is that of a retrenched welfare state and that a re-strengthened welfare state is integral to a just transformation. Among key demands are increased spending on education, including the current transition and retraining support, strengthened active labour market policies and unemployment insurance, and state support to transition-regions. Many actors emphasise a need to uphold collective bargaining and decent working conditions in transitioning sectors. Distributional aspects in everyday life are regarded as critical, even more than risks for unemployment. A recurring view is that it should be possible for all to participate in the transformation, regardless of income or place of residence. Among the raised proposals are investments in green infrastructure, including public transportation, subsidies of green technologies, and support to low-income groups and rural populations.

For environmental CSOs, the central justice dimension is global. Actors want to strengthen rather than restore Swedish climate goals. Legitimacy is thus viewed as essential: ‘That is why a just transformation is so important. It’s about bringing people along’, argues a respondent of a large environmental CSO. The global perspective is combined with a local focus: ‘We want the transformation to be run locally […] It’s a question of democracy’, continues the same respondent. While perceptions of scale and scope thus differ, many proposals resemble those raised by trade unions, including on education and security under transformation. The focus, however, is different. Environmental CSOs target consumption, including flying and meat consumption. While environmental CSOs support the industrial transition, emphasis is put on needs to minimise energy use and resource extraction.

Samí actors and the youth climate movement stand out in visions of a deeper transformation. ‘We are not just interested in a green transition. We want systemic change. […] Nature has become an aggression point, something used for extraction and capitalisation’, argues a Samí respondent. A just transformation is perceived as a shift from colonial and capitalist exploitation: ‘I am talking about exploitation of both people, nature and ecosystems. If we continue to exploit species and ecosystems, if we sacrifice them on the altar of climate transformation, then we will not create a sustainable system’, says a respondent of the youth climate movement.

The CSO representing rural interests raises another key dimension of a just transformation: between centre and periphery. Among the central demands are equal opportunities to participate in rural regions, including investments in infrastructure and public services, access to finance for SMEs, and to benefit from a larger share of the values extracted from rural areas.

The rural–urban divide is highlighted by almost all actors. One reason is arguably the relatively strong support in rural areas for current climate policy changes. ‘We have members who are very dedicated to the climate transformation, but also those who are very concerned about the ability to work and live here’, says the respondent of the rural CSO. Reflecting on the causes of the policy rollback, the respondent argues that support in rural areas stems from a sense of neglect and growing injustices, including declining infrastructure and public services: ‘It is not really about the climate transformation, but it takes that expression. High diesel price is not the sticking point, but it becomes the symbol of it all’.

Questions of justice are not as familiar for business actors. Some even refrain from using the word ‘just’. However, issues of a just transformation seem to be of increasing concern. While core demands are focused on a smooth industrial transition—faster permit processes, investments in fossil-free power production, new infrastructure and education of skilled labour—these are increasingly complemented with other requests, such as investments in social structures, including housing and welfare services in regions undergoing rapid transformation: ‘You can’t build a steel factory in [the city of] Boden if there are no schools, buses, football teams, hockey teams, theatres.’, says a respondent of a large employers’ organisation.

Another key issue is the worry that eroding legitimacy will derail the transition towards a fossil-free Sweden: ‘We will not get acceptance for a climate transformation […] if there are groups who think that they carry a bigger burden, and that they or society don’t benefit’, argues a respondent of a large employers’ organisation. ‘If [the transformation] is not perceived as just and legitimate, we will not be able to proceed as fast as we need’, states an industry respondent. Solutions from business range from subsidised green technology to new redistributive policy tools. ‘What is needed is distributional policy. That you… identify groups where consequences are high and make sure to compensate those groups’, argues a respondent of one employers’ organisation.

The respondent representing SMEs stands out among business actors, preferring the term ‘level playing field’ to ‘just’. However, raised concerns have clear justice dimensions: lack of capacity to handle bureaucracy following the European Green Deal, and lack of investment resources available to big business. ‘Justice is also to acknowledge that things look different, for instance between large and small firms. […] And we see that policies are developed from the perspective of large corporations’, argues the respondent.

### Financing a just transformation and the role of the state

A broad majority of actors argue that a rapid and just climate transformation requires increased public finance. A common perspective is a perceived need for a more active state, engaging in risk sharing and securing necessary funding, including for investments in infrastructure, energy production, education and social structures.

One option for increased funding is taxation. On such issues, however, we find little convergence. Environmental CSOs focus on the polluters pay principle including phasing-out fossil fuel subsidies, or increasing taxes on environmental bads while lowering them on environmental goods. Trade unions are more concerned about distributional effects. While subscribing to the polluters pay principle, blue-collar unions also argue for raised taxes on capital, wealth or high-income groups, to finance both re-strengthened welfare and a just climate transformation. Business actors are sceptical towards raised taxes in general, preferring tax cuts on environmental goods, but commonly accepting the polluters pay principle if measures harmonise with EU policies.

In order to raise necessary funding, many actors instead discuss a revision of the Swedish fiscal policy framework, allowing deficit funding for public investments. The framework has fostered budget discipline since the 1990s, including a surplus target and an expenditure ceiling, contributing to one of the lowest public debts in the EU (Calmfors 2023). On this point, a considerable shift in positions can be observed among key actors, including all trade union confederations, a number of CSOs, and—perhaps more surprisingly—large business organisations (Daunfeldt and Frycklund 2024): ‘This is a transformation we need to do… Therefore, it is reasonable to increase the national debt a bit, and pay it back later’, says one industry respondent. Business, and in particular trade union respondents, describe a long-term ‘investment debt’, involving infrastructure in general: ‘This is exactly what we have not done in Sweden for many years… We are so far behind in many respects and we need to do so much and so fast […] There is no reason for us to pay down on a national debt which is almost non-existent’, argues the respondent of a trade union confederation. A need for a revised fiscal framework has also been raised by expert committees, including the Swedish Climate Policy Council (2024).

### Environmental conflicts: The depth of a just transformation

The most critical tensions among the respondents concern the depth of the climate transformation, including environmental and land use conflicts.

One contentious issue concerns the establishment of wind farms. To resolve localisation conflicts, a broad majority of actors argue that municipalities or communities should receive compensation and take part of benefits deriving from wind power production. Such convergence, however, is absent on other issues. One example is forestry, where forest industry actors diverge significantly from Samí and environmental CSOs. Cleavages include production volumes, harvesting methods and carbon sinks, where Samí actors and environmental CSOs argue for continuous cover forestry, i.e. avoiding forest clearcuttings that damage biodiversity, and more protected areas. Some convergence, however, can be found on ideas to support owners who protect biodiversity and contribute to carbon sinks.

The tensest conflicts relate to Northern Sweden, where the green industrial transition—notably iron and steel industry, but also battery factories—has opened new commodity frontiers for extraction of natural resources, including wind power and infrastructure. Environmental CSOs fear that the industrial transition will be carried out at the cost of biodiversity and human rights. ‘There are a vast number of interests colliding. And we are in the middle of the shit. Because we try to defend indigenous people and environmental rights. And simultaneously we want to achieve a climate transformation’, comments one established environmental CSO. Among Samí respondents, the combination of wind power, grids, intense forestry and in particular mining, are viewed as existential threats: ‘I am afraid we are screwed […] They will run over us without pardon. They don’t give a shit about [human rights]’.

Such views stand in stark contrast to those of business actors, demanding faster permit processes. While most respondents recognise the potential conflicts, few have articulated ideas for how they may be resolved. Many industrial actors argue that trade-offs are unavoidable: ‘If we are to succeed we need to double the power system. Which means more production, more grids. And the places already taken are those creating least problems. Which means we will only get more opposed interests. And conflicts’, as one industry respondent puts it. Trade unions share similar views, in particular regarding energy production and transition minerals. While many unions argue that mining in Sweden is more just than relying on foreign extraction, positions also include a critique of extraction for profit alone and demands on a more circular economy.

Almost all actors share a view that politics have shied away. ‘In one way or another, politics have to deal with these conflicts. They cannot simply duck.’, says one respondent of an employers’ organisation. Many actors emphasise a need for strengthened dialogues and local participation, in particular in early phases of processes. A recurrent view is that a larger share of the value extracted from resources should benefit municipalities and communities. Unlike the case of wind power, however, there is little convergence on how and in what cases such mechanisms should be established. The most articulate—and perhaps radical—proposal comes from Samí respondents and the youth climate movement: giving legal standing and inherent rights to non-human life: ‘We need a paradigm shift in the right to exploit nature. Corporations have rights. But nature has no rights in the Swedish legal system. […] What is needed is strengthened legal rights […] But also a voice in decision processes. It could be in the form of appointed Nature Guardians’, says a respondent of the youth climate movement.

These diverging views intersect with a deeper cleavage: on the depth of a just transformation. All business actors express a firm belief in green growth, either in the ability to decouple growth from environmental impacts, or as the only viable path. Environmental CSOs are decisively more sceptical and lean towards a growth-critical or growth-agnostic perspective, but refrain from arguing for degrowth: ‘We see enormous problems with the extensive resource extraction today. But you also have to consider political realities to achieve something’, as one environmental CSO puts it. Samí actors and the youth climate movement stand out by taking a clear position for a transformation away from growth as such: ‘We don’t believe in growth. For us growth is that nature prosper. […] That you don’t consume more than you need. But this is contrary to the whole capitalist structure we live in’, says one Samí respondent. The picture among trade unions is diverse. While many respondents, in particular industrial and white-collar unions, distance themselves from degrowth, we also find an emerging agnostic perspective: ‘I would put myself in-between [green growth and degrowth]. I don’t think people become happy because we have a high GDP’, one trade union confederation respondent argues.

### Possible compromises, alliances and neglected interests

In summary, while deep cleavages exist on the depth of a just climate transformation, our material reveals new and coinciding interests between a majority of the respondents. This includes strong demands for political leadership, widespread dissatisfaction with the current dismantling of climate policies, and a view that stated climate goals should be kept or even strengthened. Maintaining Sweden’s status as a frontrunner and increased state responsibilities to achieve such goals are underlined as key, including financial reforms and new policy tools to ensure what actors perceive as a just transformation.

Table 3 summarises key policy demands, sorted according to support. Broad support can be found behind: (i) calls for political leadership; (ii) maintaining climate goals and Sweden as a frontrunner; (iii) a more active and interventionist state; (iv) risk sharing and public investments in energy, infrastructure, education and social structures; (v) new policy tools to ensure legitimacy, participation and redistribute the costs and benefits of a climate transformation; and (vi) structural financial reform, allowing increased public investments and expenditures.

Many respondents also express a sense of a shared direction, including a willingness to cooperate with other actors. As one industrial organisation puts it: ‘We [all] want a good climate transformation for Sweden. Which gets legitimacy and acceptance, where we create jobs, welfare and energy independence. All organisations want this.’

From one perspective, such statements downplay underlying contradictions. A majority of the actors, however, also share broad problem descriptions, including perceived lack of political leadership, a worry that unequal circumstances delegitimise the transition and that such problems have been used by political forces feeding ‘culture war’ and ‘climate denial’. Many actors share a desire to counteract such developments.

The sense of coinciding interests is particularly strong among trade unions and business organisations. ‘Large parts of business are co-players in this, not opponents’, states the respondent of one large trade union actor. ‘We quarrel in the traditional negotiations. But on these issues, we share a lot’, argues one industrial employer organisation.

Business and trade union actors recurrently refer to the Swedish model. It is described as a ‘superpower’ and viewed as a potential basis for achieving a just transformation and, even, for a ‘green’ Swedish model. Our material indicates ongoing dialogues between trade unions and business actors on agreements to ensure conditions for a just transformation, to some extent also involving environmental CSOs. However, ‘the condition for a big deal is not there yet’, says a representative of a central trade confederation and refers to current political conditions and diverging views among leading business interests. ‘If it was just us [in the basic industries] we could do it’, comments one employer organisation. Instead, diverse dialogues currently evolve at sectoral levels. ‘One idea is to build on the industry roadmaps [towards a fossil-free Sweden] and work out common agreements from them’, says a trade union representative. The biggest hurdle, however, is the perceived absence of the third part in the constellation: the state. ‘We are pretty damn good at structural transformations in Sweden. […] So what we have to do is to bloody tell the politicians that they have to put themselves together’, says a respondent of a trade union confederation.

The relations between business and trade unions, and environmental CSOs and Samí actors, are more complex. Conceptually, the idea of a green Swedish model resonates broadly among environmental CSOs: ‘We really want something like that. … The more actors that can work on a shared agenda, the easier it will be’, as one respondent says. The youth climate movement agrees: ‘A green Swedish model? It is a gorgeous idea. If the core principles are dialogue, respect for each other’s rights, and a strive towards compromises’.

Simultaneously, a critical question is how the original Swedish model could be extended to actors and interests other than trade unions and business organisations. Samí respondents raise substantial fears that compromises among dominant actors will ignore the rights of indigenous people and nature itself. The question also concerns the institutional form of agreements. The Swedish model is based upon collective bargaining between labour and capital. ‘I will meet with [an environmental CSO] today. We gladly join forces to put pressure on politics. But a deal is something else’, says the respondent of one trade union confederation. ‘If you make deals you must be able to take responsibility. That is how collective bargaining works. But for nature… It is not how the system looks like’, reasons a respondent of a large employers’ organisation. Interestingly, Samí actors and the youth climate movement agree on this point, arguing that a deal including environmental interests may have to be constructed in other ways, including strengthened legal rights for nature and indigenous people.

## Discussion and conclusions

This study has analysed perceptions of a just climate transformation among business, trade union and civil society actors in Sweden. In a period of governmental rollback of climate policy, key actors oppose this break. Building on Sweden’s frontrunner position and traditions of the Swedish model, they not only remain ambitious, but advance their positions and open for collaborations with new actors. To what extent this holds true over time, and what the implications might be, call for further studies.

While the actors differ, in particular on the depth of a just climate transformation, they share interests for a set of critical political demands (see Table 3). This includes both managerial and structural reforms, pointing towards a broader and more interventionist environmental state, and a partly renewed and extended welfare state, backed up by a structural financial reform.

Such structural change would follow contemporary trends towards more active state interventions (Gabor 2023) to handle the interlinked challenges of climate transformation and growing social and democratic tensions. Furthermore, the coinciding interests illustrate an interesting dynamic, whereby calls for a just transformation among CSOs merge with a competitive pressure towards decarbonisation among industries. Actors are glued together by concerns about the climate policy backlash and a desire to restore Sweden as a frontrunner. This contrasts with the situation not so long ago, when key actors were less engaged or took other positions. And again, the frontrunner position seems to strengthen resistance to rollback and thus provides stability in times of weakened political leadership. The dynamics may be compared with how certain states and actors responded in the US during the first Trump administration and is arguably of wide interest today.

Many actors also express a wish to restore social cohesion and the Swedish model in a wider sense, counteracting perceived reactionary countermovements by raising an appealing vision of a brighter and less conflictual future. In particular, trade unions and business organisations elaborate on shared interests in terms of a green or reinvigorated Swedish model. Such a vision of a broad agreement towards a just climate transformation seems to resonate widely among the actors. Simultaneously, actors differ, in particular in their views on environmental conflicts and depth of a just transformation. On these issues, the analysis highlights the risk that a compromise might ignore such tensions, a risk which calls for further inquiry.

What are the prospects for realising a green Swedish model? The answer depends on many factors, including the interests and power relations between the investigated actors, but also on key actors not included in this study: political parties. Compared to the transformational change underlying the emergence of the Swedish welfare state, several observations can be made. While coinciding interests for change exist between trade unions, civil society organisations and business elites, the context today is different. The labour movement in the 1930s was a mass movement with close links to a political party with large electoral power. Today, trade unions are weaker, and the political landscape is different, whereas capital interests and reactionary countermovements are stronger, i.e. power relations are different. While industrial actors may be interested in a green Swedish model, the same is not necessarily true for all business interests. The transition towards a fossil-free welfare state may be attractive for the Swedish steel industry and large manufactures but not necessarily for SMEs. Other capital interests may simply be uninterested in a just climate transformation.

Simultaneously, broader developments seem to push for change. A key ingredient in the 1930s was the structural shift in economic policy. Interestingly, such a shift may be underway. The financial burden of decarbonisation and social challenges have been accompanied by geopolitical instability and perceived needs for military rearmament. In Sweden, an increasing number of actors call for reforms of the fiscal framework, including leading economists (Calmfors 2023) and the most influential business organisation (Daunfeldt and Frycklund 2024). While political elites so far resist, this might lead to structural change. A shared agenda among trade unions, environmental CSOs and industrial actors may constitute a force with considerable leverage over political parties. The agenda may not constitute the deep transformational change that some actors desire, but could imply deeper cuts in emissions, a reinvigoration of the welfare state, and a broader participation and support for the transformation towards a fossil-free welfare state. Following Eckersley (2021), this could be described as ‘critical problem-solving’, i.e. a step-wise process where structural reforms widen the options for further change.

To conclude, our study has identified new and coinciding interests among key Swedish actors. Paradoxically, the rollback in Sweden has broadened such coinciding interests. The shared interests may provide building blocks of a green Swedish model, and could further emerge as an alternative route compared to the current rollback. The Swedish frontrunner position may be unique, but the pattern of backlash is certainly not. While actors and issues differ depending on context, some key elements remain the same. This arguably could include shared interests in public investments, new redistributive tools, a more interventionist state, and financial reforms and political leadership to enable such policies.

## Supplementary Information

Below is the link to the electronic supplementary material.

Supplementary file1 (PDF 196 KB)

## Data Availability

The participants of this study have not given written consent for their data to be shared publicly, so raw data is not available.

## Footnotes

1 We use the UN definition of CSOs: ‘non-State, not-for-profit, voluntary entities formed by people in the social sphere that are separate from the State and the market’. https://www.ungpreporting.org/glossary/civil-society-organizations-csos/
