# Supplementary material for: Exploring a green Swedish model: Coinciding and contradictory interests on a just climate transformation in Sweden
Source: Ambio. 2025 Feb 19;54(7):1237–49. doi: 10.1007/s13280-025-02144-6 (PMC12133620; doi:10.1007/s13280-025-02144-6)
Supplement: Supplementary file 1 — Supplementary file1 (PDF 196 KB) [file 13280_2025_2144_MOESM1_ESM.pdf]

Ambio

Supplementary Information

This supplementary information has not been peer reviewed.

Title: **Exploring a green Swedish model: Coinciding and contradictory interests on a just climate transformation in Sweden**

Appendix S1: Interview methods table

Actor	Date	Source	Format	Length	Method	Status
**Category 1: Trade unions**						
Trade union confederation 1	29/11 2023	Sample frame	Semi-structured	90 min	Tape	Done
Trade union confederation 2	7/12 2023	Sample frame	Semi-structured	110 min	Zoom	Done
Trade union confederation 3	23/11 2023	Sample frame	Semi-structured	90 min	Tape	Done
Trade union 1	19/12 2023	Sample frame	Semi-structured	120 min	Tape	Done
Trade union 2	12/1 2024	Sample frame	Semi-structured	90 min	Tape	Done
Trade union 3	29/11 2023	Sample frame	Semi-structured	90 min	Tape	Done
Trade union 4	11/1 2024	Sample frame	Semi-structured	90 min	Zoom	Done
Trade union 5	14/11 2023	Sample frame	Semi-structured	70 min	Tape	Done
Trade union 6	24/11 2023	Sample frame	Semi-structured	90 min	Zoom	Done
Trade union 7	12/12 2023	Sample frame	Semi-structured	120 min	Zoom	Done
Trade union 8	23/11 2023	Sample frame	Semi-structured	105 min	Zoom	Done
**Category 2: CSOs[1]**						
Environmental CSO 1	8/11 2023	Sample frame	Semi-structured	70 min	Tape	Done
Environmental CSO 2	29/11 2023	Sample frame	Semi-structured	90 min	Zoom	Done
Environmental CSO 3	29/11 2023	Sample frame	Semi-structured	120 min	Zoom	Done
Environmental CSO 4	8/12 2023	Sample frame	Semi-structured	120 min	Zoom	Done
Environmental CSO 5	Declined	Sample frame				
Environmental CSO 6	No response	Sample frame				
CSO 1[2]	1/12 2023	Sample frame	Semi-structured	120 min	Zoom	Done
CSO 3	24/11 2023	Sample frame	Semi-structured	90 min	Zoom	Done
CSO 3	20/12 2023	Sample frame	Semi-structured	90 min	Zoom	Done
CSO 4	No response	Sample frame				
**Category 3: Industry and employers' organisatioı**						
Central employers' organisation 1	4/12 2023	Sample frame	Semi-structured	90 min	Tape	Done
Central employers' organisation 2	9/1 2024	Sample frame	Semi-structured	120 min	Zoom	Done
Employers' organisation 1	5/12 2023	Sample frame	Semi-structured	110 min	Tape	Done
Employers' organisation 2	5/12 2023	Sample frame	Semi-structured	110 min	Tape	Done
Employers' organisation 3	7/1 2024	Sample frame	Semi-structured	120 min	Zoom	Done
Employers' organisation 4	No response	Sample frame				
Industry organisation 1	24/11 2023	Sample frame	Semi-structured	115 min	Zoom	Done
Industry organisation 2	6/12 2023	Sample frame	Semi-structured	115 min	Tape	Done
Industry organisation 4	30/11 2023	Sample frame	Semi-structured	100 min	Tape	Done
Industry organisation 5	9/11 2023	Sample frame	Semi-structured	70 min	Tape	Done
Industry organisation 6	7/12 2023	Sample frame	Semi-structured	90 min	Tape	Done
Industry organisation 7	14/12 2023	Sample frame	Semi-structured	90 min	Zoom	Done
Farmers organisation	7/12 2023	Sample frame	Semi-structured	70 min	Zoom	Done

[1] We use the UN definition of CSOs: 'non-State, not-for-profit, voluntary entities formed by people in the social sphere that are separate from the State and the market'. https://www.ungpreporting.org/glossary/civil-society-organizations-csos/

[2] No formal CSO, but critical for indigenous people representation.

Appendix S2: Questionnaire for semi-structured interviews

The following questionnaire is used for the semi-structured interviews. Observe that questions were adjusted depending on type of actor, i.e. depending on whether the interviewee represents a business organisation, a trade union, an environmental CSO, and so on.

General questions about climate goals and climate transformation

- Is it important that Sweden reaches decided climate goals and acts in accordance with the Paris Agreement? Why/why not?
- Is it important that Sweden is in the forefront on the climate transformation? Why/why not?
- How do you reflect upon recent changes in climate policy in Sweden and the EU?
- What main challenges/opportunities do you see with the climate transformation?
- What political decisions do you consider most important in order to reach Sweden's climate goals?

General questions about a just climate transformation

- Is it important that the climate transformation is perceived as just? Why/why not?
- What do you consider as a just climate transformation?
- Economic inequalities have grown in Sweden and other countries. Do you think this affects the abilities to achieve a just climate transformation?
- Does a just climate transformation have other goals than climate goals?

- What political decisions do you consider most important to enable a just climate transformation?

Just climate transformation: Production and labour market perspective

- How do you think the climate transformation will affect employment and the labour market in Sweden?
- Is it important that the climate transformation does not lead to increased unemployment?
- Should all jobs and corporations be ‘saved’?
- What political decisions are needed in order to enable a just transformation on the labour market, for labour and business?
- Possible follow ups on education, labour market policy, social security, and needs for business.

Just climate transformation: Consumer and citizen perspective

- The climate transformation may affect citizens, including through increased living costs, needs to invest in new technologies, or needs to change lifestyles. In what ways do you believe the climate transformation will affect citizens?
- What are the implications of people having different capabilities to handle such costs and participate in the climate transformation?
- What are the implications of people having different responsibilities for GHG emissions?
- How should costs for the climate transformation be distributed in order for it to be just, and do you see a need for political decisions to achieve this?

- From the opposite perspective: What benefits and economic gains with a climate transformation do you see, and is there a need to make sure that those gains and benefits are distributed in a just manner?

Financing a just climate transformation

- Do you think a just climate transformation in line with Swedish and international climate goals will call for increased public spending and investments?
- If so: Does such increased need for public spending and investments call for new financial policies?
- Does a just climate transformation call for tax reforms, and in that case what kind of tax reforms?

Environmental conflicts, land conflicts, economic growth and sufficiency

- In what ways could the climate transformation collide with other environmental goals, local interests, or human rights?
- What is needed in order to resolve such conflicts?
- According to the IPCC new technology and efficiency measures will not be enough to reach the goals of the Paris Agreement. Other measures will also be needed, including reduced energy and resource use, and changes in lifestyles and consumption. Do you agree with this conclusion?
- How could such measures be achieved in a just way?
- Scientists sometimes distinguish between those who believe that green growth can take us to the climate goals, those who argue for degrowth, and those who argue that BNP as such is not the interesting measure, but rather the content of the economic development. What is your take on this?

A just climate transformation: Participatory justice

- We have discussed distributional justice. What is needed in order for the climate transformation to be just from a democratic perspective, both on the labourmarket or in society at large?
- Are there need for specific measures to strengthen or ensure democratic participation?

Contradictory and coinciding interests

- What key conflicts in society do you see when it comes to achieving a just climate transformation in line with Swedish and international climate goals?
- What conflicts do you see between your own organisation and other actors?
- What common interests do you see between your organisation and other actors when it comes to achieving a just climate transformation?
- Could such common interests serve as a foundation for some kind of agreement or compromise to enable a just climate transformation?
- Is there something you would like to add, or something you had expected us to ask?

Appendix S3: Coding process

All interviews were transcribed using a Whisper-based model. The transcripts were pseudonymised, and the participating actors given attribute codes, indicating category, type of actor, and the role of the interviewee in the organisation. The coding was then performed in two stages.

In the first stage, coding was done by combining two main methods: (1) a structural, conceptual-based coding, mainly deriving from the themes and sub-themes raised in the interviews (such as 'Sweden's climate goals', 'just transformation, distribution of burdens', 'just transformation, financing', 'environmental conflicts'), and (2) a descriptive, more in-vivo-based coding, focusing on the respondents perceptions of the discussed themes. Particular attention was put on perceived challenges, conflicts or enemies, on the one hand, and perceived opportunities, solutions or allies, on the other hand, categorising these using different types of coding. For certain themes it was possible to use magnitude coding, i.e. to categorise the respondent's views into 'yes', 'no' or other distinct categories.

In the second, analytical stage, the derived codes were condensed and compared between the different types and categories of actors, in search of conflicts and coinciding interests, including possible alliances and bases for compromises.
